# Parosteal osteosarcoma of the temporal bone: Case report

**DOI:** 10.1016/j.radcr.2023.07.038

**Published:** 2023-08-01

**Authors:** Hajra Idrees, Raza Zarrar, Bilal Mujtaba

**Affiliations:** a Department of Musculoskeletal Radiology, University of Texas MD Anderson Cancer Center, 1515 Holcombe Blvd, Houston, TX, USA; b Department of Internal Medicine, Baptist Hospitals of Southeast Texas, 3080 College St, Beaumont, TX, USA

**Keywords:** Temporal bone parosteal osteosarcoma, Computed tomography, Magnetic resonance imaging

## Abstract

Parosteal osteosarcomas (POS) are well-differentiated low-grade malignant sarcomas that are located on the surface of the bone. POS of the temporal bone is exceptionally rare, with less than a hand full of cases present in modern literature. Here, we report a POS of the temporal bone found incidentally and with an uncharacteristic presentation. We also review the unique imaging and histopathological findings of this entity and discuss why developing a broad differential diagnosis and proceeding with early intervention are considered imperative in this disease.

## Introduction

Parosteal osteosarcoma (POS) is a rare tumor that arises from the cortex of the bone that was first described in 1951 [1]. The first case of POS in the craniofacial region was reported 10 years later [2]. POS of the temporal bone is exceeding rare with only 4 cases found in modern literature [3]. Presentation of POS can range from an asymptomatic mass to a severely painful lesion causing reduced joint mobility, skin ulcerations, and lymphadenopathy [4]. A broad differential diagnosis is essential which includes benign and malignant tumors, metastatic lesions, and sequalae of injury [3]. Clinical features, laboratory testing, imaging, and histopathological examination are all used to reach a diagnosis [2]. Computed tomography (CT) scan and magnetic resonance imaging (MRI) are the preferred imaging modalities, due to their ability to diagnose POS more accurately. Characteristic features of POS on imaging include sunburst appearance and the so-called string sign. Both CT scan and MRI are used for clinical staging of the tumor [2,3]. CT scan is used initially due to its faster image acquisition, differentiates benign from malignant lesions based on their architecture, and rules out metastasis [3,4]. MRI is used to rule out recurrence of malignancy, bone marrow infiltration, or any soft tissue component of the lesion [5]. MRI can also help distinguish low from high-grade variants of POS [6]. MRI is the preferred imaging modality for dedifferentiated parosteal osteosarcoma (DPOS) and its response to treatment [5]. A definitive diagnosis of POS is made only on histological examination with the most widely recognized pattern called “streamer pattern” [7]. Treatment of POS is prophylactic excision of the lesion. Resected low-grade POS offers a favorable prognostic profile [2]. In case the lesion is high-grade or DPOS on postsurgical histology, further resection with wider margins and adjuvant chemotherapy can be performed [8].

## Case presentation

A 28-year-old male with a medical history of migraine disorder presented to the emergency department with pain and swelling located in the right mastoid area. The patient had been experiencing these symptoms for several months, but recently noticed a sudden increase in pain and the size of the swelling. An urgent CT scan identified a 3 cm right temporal bone mass. It was at this point that the patient was referred to our institution for further management.

**Image 1:**
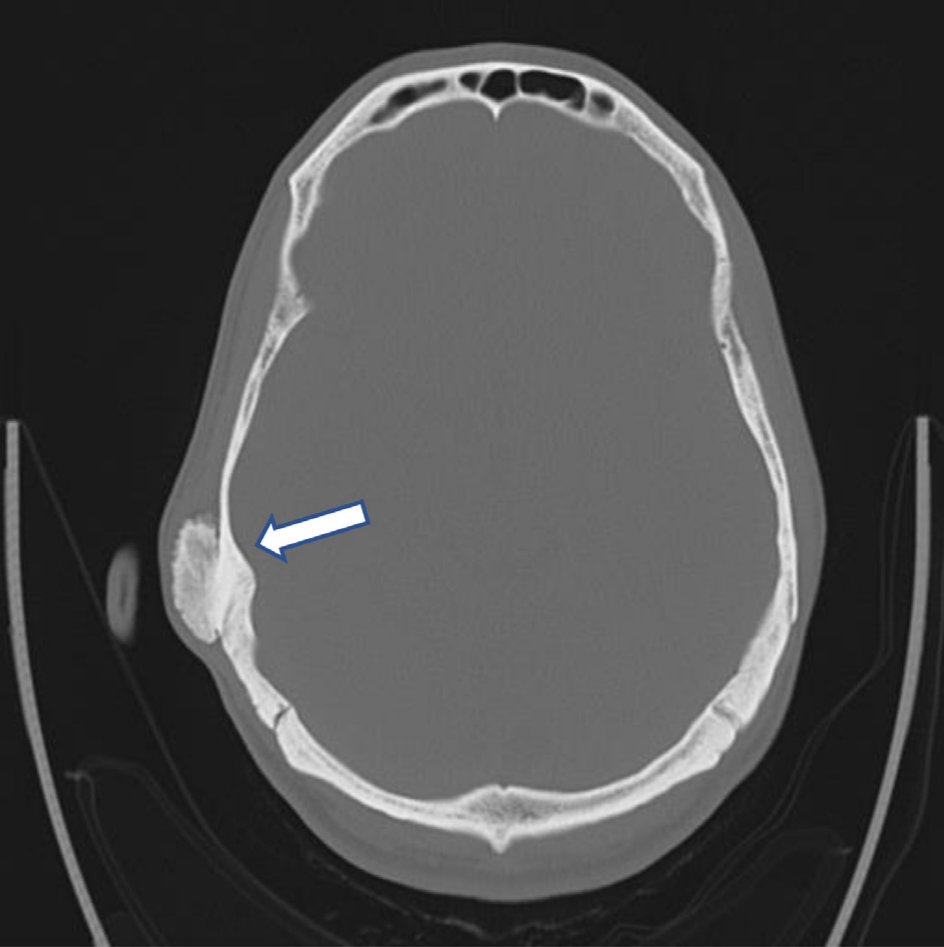
Noncontrast CT shows a benign-appearing hyperostotic lesion measuring 3.0 × 3.0 × 1.1 cm (white arrow) involving the outer table of the right calvarium. There did not appear to be an intracranial component, although some focal bone-like thickening was present in the area. There was no evidence of extracranial soft tissue swelling or mass adjacent to the mastoid bone.

The mass was suspected to be an osteoma of the temporal bone; however, the differential diagnosis also included osteochondroma and chronic sequela of prior trauma. CT imaging without contrast confirmed the lesion to be a benign-appearing hyperostotic lesion measuring 3.0 × 3.0 × 1.1 cm involving the outer table of the right calvarium (Image 1). There did not appear to be an intracranial component, although some focal bone-like thickening was present in the area. There was no evidence of extracranial soft tissue swelling or mass adjacent to the mastoid bone. The pain subsided with the help of analgesics, a referral was made to the head and neck clinic, and the patient was discharged.

**Image 2:**
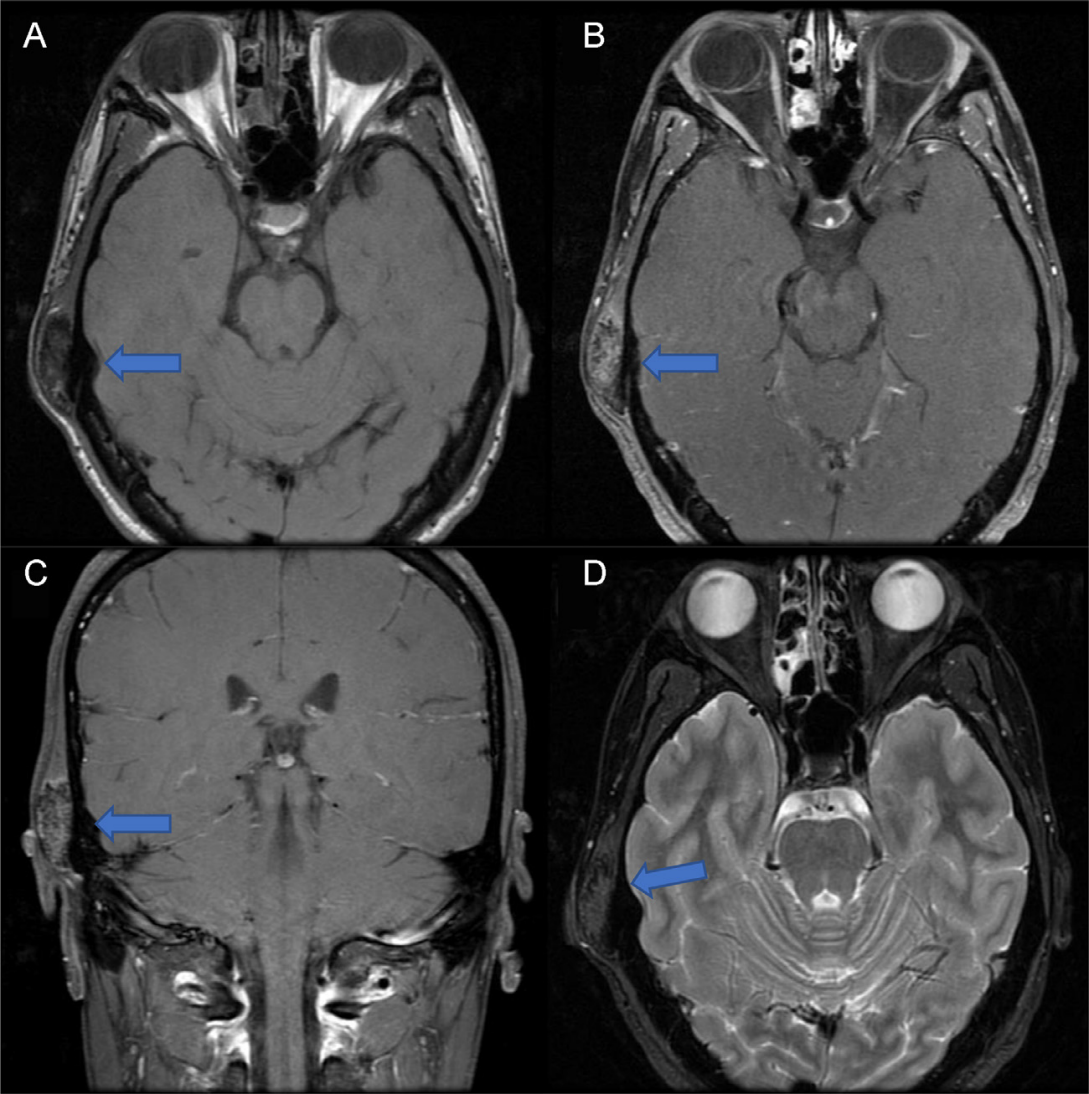
(A-D) MRI with (A, C) and without (B) contrast showed a densely calcified mass measuring 3.6 × 1.5 × 3.0 cm (blue arrows) that involves the inferior aspect of the squamosal portion of the right temporal bone and is located directly superior to the petrous portion. The lesion has a low T1 (A-C) and T2 (D) signal intensity which originates from the outer table and extends into the soft tissues with mild patchy internal enhancement. Thickening of the inner table of the calvarium is present in the same region with no abnormal signal intensity or enhancement. There is no soft tissue component present beyond the bone. No dural involvement or any other intracranial structures. A small amount of fluid in the right mastoid air cells is present. Mucosal thickening present in the right ethmoid air cells.

The patient went through an extensive clinical evaluation at the head and neck surgery department. Considering the new onset pain and subjective increase in the size of the mass, the decision was made to proceed with excision. A plan was made to perform a right-sided mastoidectomy with possible fat graft and temporalis flap. However, before proceeding with the surgery an MRI was performed to rule out any soft tissue component of the mass. MRI with and without contrast (Image 2A and B) showed a densely calcified mass measuring 3.6 × 1.5 × 3.0 cm that involves the inferior aspect of the squamosal portion of the right temporal bone and is located directly superior to the petrous portion. The lesion has a low T1 and T2 signal intensity which originates from the outer table and extends into the soft tissues with mild patchy internal enhancement. Thickening of the inner table of the calvarium is present in the same region with no abnormal signal intensity or enhancement. There is no soft tissue component present beyond the bone. The lesion does not involve the dura or any other intracranial structures. There is however a small amount of fluid in the right mastoid air cells. Lastly, there is mucosal thickening present in the right ethmoid air cells. Laboratory investigations were performed before the surgery and were found to be within normal limits. These included a complete blood count with differentials, basic metabolic panel, thyroid stimulating hormone, and free T4.

To remove the mastoid mass, the patient underwent a right mastoidectomy followed by grafting with the help of an autologous soft tissue harvested from the abdomen. Additionally, a craniectomy with cranioplasty using a titanium mesh was performed. The patient's pain improved and was discharged on oral analgesics that were to be taken only as needed. Postsurgical pathologic examination demonstrated a fibro-osseous lesion, composed of well-formed trabeculae of woven bone surrounded by a proliferation of spindle cells. Additionally, mild atypia was present, with infiltration into the skeletal muscle. The tumor extended to the edge of the tissue. The biopsy results were found to be consistent with a POS of the temporal bone

## Discussion

**Fig. 1:**
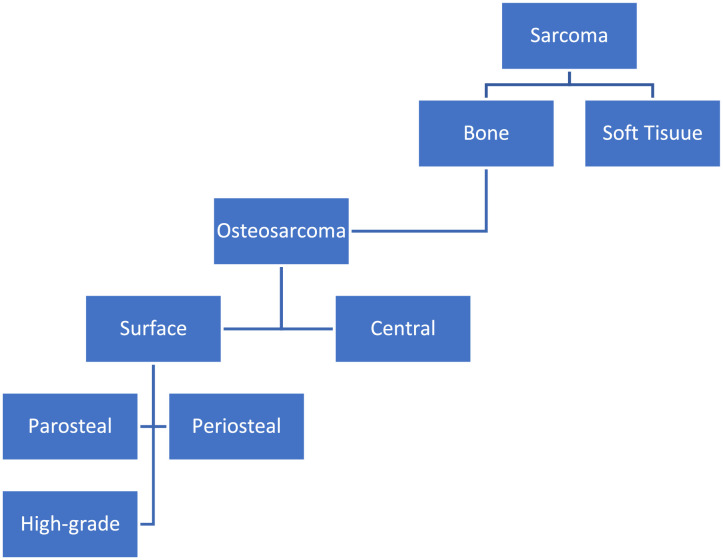
Classification of sarcoma [3].

POS is a rare low-grade tumor arising from the outer layer of the cortex of the bone. Geschickter and Copeland describe this tumor for the first time in 1951 [1]. In 1961 the first POS in the craniofacial region was reported [2]. Osteosarcoma is a type of sarcoma; further subclassification is done based on its location within the bone and histological features (Fig. 1) [3]. POS is the most well differentiated low-grade variant, when compared to the other surface osteosarcomas. POS originates from the outer periosteum, while the remaining 2 subtypes arise from the inner part of the periosteum.

POS is an uncommon tumor with location in temporal bone of the skull being exceeding rare with only 4 cases reported in modern literature [3]. POS commonly presents as a slow growing and painless mass on the surface of the bones that was similar to our case. However, one notable difference in the presentation of our patient was that the lesion was severely painful. Additionally, POS can present with lymphadenopathy, decreased range of motion in the adjacent joint, mucosal ulceration, and dull pain [4]. POS are found more commonly in females with the mean age of presentation being in the third decade of life [6].

POS has a very broad differential diagnosis that includes benign and malignant tumors, metastatic lesions, sequalae of injury and adequate testing to eliminate these possibilities is required. Benign lesions include osteoma and osteochondroma. However, primary and secondary malignant lesions are of greater concern since these can alter the treatment and prognosis for the patient [3]. Most notably osteogenic sarcoma is a more aggressive malignancy and therefore hold a worse prognosis when compared to POS [9]. Other primary malignant lesions include chondrosarcoma, high-grade, and periosteal surface osteosarcoma [3]. Clinical features, laboratory testing, and imaging studies can be used to differentiate POS from other entities, however like other malignancies the definitive diagnosis can only be achieved by histopathological analysis [2].

Imaging is an essential part of investigation for POS. Imaging modalities include X-ray, CT scan, and MRI. CT scan and MRI are preferred over conventional radiography, due to their superior ability to diagnose POS. Our patient underwent a CT scan (Image 1) without contrast as part of initial imaging for diagnosis and MRI (Image 2A-D) was performed to rule out any soft tissue component before surgical excision of the mass. Both CT scan and MRI can be used for clinical staging of the tumor. Radiological features of POS of the skull include round, sessile, lobulated, exophytic bone growths arising from the external cortex of the bone. These lesions form the so-called “sunburst pattern” as the base is denser compared to the surrounding area [3]. Another characteristic feature on imaging is the so-called “string sign” which can be visualized in approximately 30% of cases. This is a thin radiolucent cleavage plane formed between the tumor and underlying normal bone [2].

CT scan is the preferred first imaging modality due to rapid imaging requisition compared to MRI [3]. CT scan can be used for preoperative evaluation since it can demonstrate the architecture of the lesion including heterogeneity, satellite nodules, and the extent of skull involvement [6]. Additionally, CT scan aided by clinical picture can help distinguish benign and malignant lesions in addition to ruling out metastatic disease [4]. Low attenuation areas on CT scan may represent dedifferentiation, however fibrosis, soft tissue entrapment, and cartilage may appear similar [6].

MRI is advised when there is suspicion of recurrence, bone marrow infiltration and rule out any soft tissue component of the lesion [5]. MRI can help distinguish histological low-grade from high-grade variant of POS. Low-grade variants will demonstrate low-signal intensity on transverse relaxation time on T1 (Image 2A, C) and T2 (Image 2D) weighted images. This is in contrast to high-grade variants which may show high T2 signal intensity areas. However, this may appear similar to benign pathologies such as fat, necrosis, inflammation, or hemorrhage [6]. MRI can also be used to locate the ideal location for biopsy of DPOS that is relatively dense compared to it lower grade counterpart. MRI is preferred imaging modality over CT scan to diagnose DPOS, since it can better distinguish the osseous lesion from fat and necrosis. MRI can help differentiate osteomyelitis of the cranial bones that can sometimes imitate osseous lesions. Lastly, MRI can also be useful in monitoring responsiveness of DPOS to chemotherapy [5].

On histopathological examination, POS is a well-differentiated and has a biphasic appearance; composed of mature trabecular bone and fibroblastic spindle cell stroma, as was seen in our case. When the paralleled trabecular bone has intervening spindle cell stroma it gives rise to the most well-known histological pattern for POS, the so-called streamer pattern [7].

Treatment of POS involves prophylactic excision of the lesion as was the case in our patient. This is not only used to acquire tissue for histopathological analysis for definitive diagnosis, but also can be the final treatment if lesion is found to be benign. Further treatment is recommended after histopathological examination confirms the grade and extent of the lesion. Fortunately, resected low-grade POS offers a favorable prognostic profile [2]. DPOS on the other hand is associated with accelerated growth, higher recurrence rate and is more likely to metastasize. DPOS requires radical surgical excision or en bloc surgical resection with a wide cut of normal bone resulting in negative margins or occasionally amputation of the limb followed by adjuvant chemotherapy. Neoadjuvant chemotherapy can be administered to shrink the lesion and possibly save the limb [8].

In conclusion, POS of the temporal bone is a rare entity. A broad differential diagnosis is essential in initial presentation. CT scan followed by presurgical MRI are considered the imaging modalities of choice. If the imaging is characteristic of an osteosarcoma, this should prompt a histopathological evaluation, since pathology is the gold standard for definitive diagnosis of this lesion. Prophylactic surgical removal is the standard of care, especially considering the malignant potential of the lesion.

## Patient consent

Complete written informed consent was obtained from the patient for the publication of this study and accompanying images.

## References

[1] Geschickter CF, Copeland MM. (1951). Parosteal osteoma of bone: a new entity. Ann Surg.

[2] Puranik SR, Puranik RS, Ramdurg PK, Choudhary GR. (2014). Parosteal osteosarcoma: report of a rare juxtacortical variant of osteosarcoma affecting the maxilla. J Oral Maxillofac Pathol.

[3] Idrees H, Zarrar R, Mujtaba B. (2023). Parosteal osteosarcoma of the skull: pathophysiological and imaging review. Eur J Radiol Open.

[4] Hewitt KM, Ellis G, Wiggins R, Bentz BG. (2008). Parosteal osteosarcoma: case report and review of the literature. Head Neck.

[5] Futani H, Okayama A, Maruo S, Kinoshita G, Ishikura R. (2001). The role of imaging modalities in the diagnosis of primary dedifferentiated parosteal osteosarcoma. J Orthop Sci.

[6] Mangla R, Mangla M, Li S, Abdelbaki A, Bansal I, Kumar A (2018). Dedifferentiated parosteal osteosarcoma of the calvaria. Proc (Bayl Univ Med Cent).

[7] Hang JF, Chen PC. (2014). Parosteal osteosarcoma. Arch Pathol Lab Med.

[8] Sheth DS, Yasko AW, Raymond AK, Ayala AG, Carrasco CH, Benjamin RS (1996). Conventional and dedifferentiated parosteal osteosarcoma. Diagnosis, treatment, and outcome. Cancer.

[9] Parmar DN, Luthert PJ, Cree IA, Reid RP, Rose GE. (2001). Two unusual osteogenic orbital tumors: presumed parosteal osteosarcomas of the orbit. Ophthalmology.

